# Robust Three-Dimensional (3D) Expansion of Bovine Intestinal Organoids: An In Vitro Model as a Potential Alternative to an In Vivo System

**DOI:** 10.3390/ani11072115

**Published:** 2021-07-16

**Authors:** Bo-Ram Lee, Hyeon Yang, Sang-In Lee, Inamul Haq, Sun-A Ock, Hayeon Wi, Hwi-Cheul Lee, Poongyeon Lee, Jae-Gyu Yoo

**Affiliations:** 1 Animal Biotechnology Division, National Institute of Animal Science, Rural Development Administration, Wanju-gun 55365, Korea; yh0415@korea.kr (H.Y.); inam7353@gmail.com (I.H.); ocksa@korea.kr (S.-A.O.); why7829@korea.kr (H.W.); pylee@korea.kr (P.L.); vetjack@korea.kr (J.-G.Y.); 2 Department of Animal Biotechnology, Kyungpook National University, Sangju-si 37224, Korea; silee78@knu.ac.kr; 3 Planning and Coordination Division, National Institute of Animal Science, Rural Development Administration, Wanju-gun 55365, Korea; hclee@korea.kr

**Keywords:** bovine, intestinal stem cells, organoid, characterisation, gene expression

## Abstract

**Simple Summary:**

The ability to recapitulate stem cells’ self-organising potential, creating three-dimensional (3D) structures of stem cells, has revolutionised various fields. Recently, in vitro 3D organoid systems are now used as alternative research tools because they serve the same purpose as in vivo systems. In the present study, we developed bovine intestinal organoids based on a 3D culture system and evaluated their characteristics. These bovine intestinal organoids, used in an in vitro model as a potential alternative to an in vivo system, hold great promise for further potential use in determining disease-modelling-based host–pathogen interactions and nutritional research for feed efficiency measurements.

**Abstract:**

Intestinal organoids offer great promise for disease-modelling-based host–pathogen interactions and nutritional research for feed efficiency measurement in livestock and regenerative medicine for therapeutic purposes. However, very limited studies are available on the functional characterisation and three-dimensional (3D) expansion of adult stem cells in livestock species compared to other species. Intestinal crypts derived from intestinal organoids under a 3D culture system from the small intestine in adult bovine were successfully established and characterised for functionality testing, including the cellular potentials and genetic properties based on immunohistochemistry, immunocytochemistry, epithelial barrier permeability assay, QuantSeq 3′ mRNA-Seq. data and quantitative reverse transcription-polymerase chain reaction. Intestinal organoids were long-term cultivated over several passages of culture without loss of the recapitulating capacity of crypts, and they had the specific expression of several specific markers involved in intestinal stem cells, intestinal epithelium, and nutrient absorption. In addition, they showed the key functionality with regard to a high permeability for compounds of up to FITC-dextran 4 kDa, while FITC-dextran 40 kDa failed to enter the organoid lumen and revealed that the genetic properties of bovine intestinal organoids were highly similar to those of in vivo. Collectively, these results provide a reliable method for efficient isolation of intestinal crypts from the small intestine and robust 3D expansion of intestinal organoids in adult bovine and demonstrate the in vitro 3D organoids mimics the in vivo tissue topology and functionality. Finally, intestinal organoids are potential alternatives to in vivo systems and will be facilitated as the practical model to replace animal experiments for various purposes in the fields of animal biotechnology.

## 1. Introduction

The ability to recapitulate stem cells’ self-organising potential, creating three-dimensional (3D) structures of stem cells, has revolutionised various fields in regenerative medicine and the fundamental study of biological processes such as growth and differentiation [1,2]. In particular, these organoid-based systems hold great promise for further potential use in determining disease-modelling-based host–pathogen interactions and nutritional research for feed efficiency measurements to improve productivity in the fields of animal biotechnology [2,3,4,5,6,7,8]. Recently, significant efforts have been made to establish a physiologically relevant in vitro system based on 3D organoids, including the small intestine and liver, in animals for practical applications, resulting in the reduction and replacement of animal experiments [9,10,11,12,13,14]. However, very limited studies are available on the functional characterisation and 3D expansion of adult stem cells isolated from livestock, compared to any other animals.

Organoids have been successfully developed from two sources of stem cells, organ-restricted adult stem cells (ASCs) from various sets of organs, such as the intestine, kidney, lung, liver, pancreas, and brain, and pluripotent stem cells, including embryonic stem cells and induced pluripotent stem cells [15]. Since the first report in 2009 [16], intestinal organoids, such as the mini-gut, have evolved as a potential alternative to in vivo models for the use of various purposes [17]. They are capable of closely replicating the structure and cellular composition of a functional native intestinal epithelium, including intestinal cell types, e.g., enterocytes, goblet cells, and Paneth cells [16,18]. Consequently, in vitro 3D organoid systems are now used as alternative research tools because they serve the same purpose as in vivo systems [19,20,21].

Cow is an economically important domestic animal species for the production of milk and meat worldwide [9]. However, cattle, as experimental animals, are highly expensive and labour intensive, particularly when sampling and separating several different tissues from slaughter [22]. Moreover, in vitro 2D culture systems such as immortalised [23] and cloned [24] intestinal epithelial cell lines have the current limitation of lacking physiological relevance and the cellular diversity being composed of the intestinal epithelium [9]. Therefore, the development of a reliable method for robust 3D culture system derived organ-restricted ASCs from various sets of organs in livestock is urgently necessary.

In the current study, we successfully established bovine intestinal organoids representing typical crypt–villus structure and intestinal epithelium. We also characterised the expression levels of several specific markers in reference to intestinal stem cells and nutrient uptake using QuantSeq 3′ RNA-Seq. data and immunocytochemistry. Furthermore, we investigated epithelial barrier function using a FITC-dextran permeability assay.

## 2. Materials and Methods

### 2.1. Experimental Designs, Animals and Animal Care

The present study was designed with the aim of investigating adult intestinal stem cells and subsequent cultivation of intestinal organoids in bovine. Hanwoo cattle (*Bos Taurus coreanae*) (>24 months old) were experimentally used. All procedures, including sample collection, and handling followed the standard operating protocols of the Animal Biotechnology Division at the National Institute of Animal Science, Korea.

### 2.2. Isolation of Intestinal Crypts and Three-Dimensional (3D) Culture System

Different jejunum fragments from the small intestine were obtained from Hanwoo cows and collected in a washing buffer containing ice-cold phosphate-buffered saline (PBS, Grand Island, NY, USA) containing 1% penicillin/streptomycin (Sigma-Aldrich, Grand Island, NY, USA). The fragments were opened longitudinally and washed thoroughly with washing buffer to remove the debris. Mucosal and submucosal layers were gently scraped off using a glass slide. The remaining muscle layer was collected into a 50 mL tube containing 30 mL of washing buffer after being cut into 3 to 5 mm pieces, repeatedly washed by shaking vigorously, and centrifuged at 300× *g* until the supernatant was clear. The collected pellet was resuspended in 25 mL of Cell Disassociation Solution (StemCell Technologies, Vancouver, BC, Canada) and incubated at room temperature for 40 min on a rocker to release the crypts. Crypts were collected after pipetting and centrifugation at 300× *g* for 5 min. The pellet was resuspended in 1 mL of intestinal human organoid medium (StemCell Technologies, Vancouver, BC, Canada), and the crypts were counted under an inverted microscope. The seeding mix composed of medium with 100–150 crypts, and Matrigel in a 1:1 ratio was prepared and then placed in the middle of a 24-well plate. The cells were returned to an incubator to polymerise the 100 μL of Matrigel dome, and after 20 min, 1 mL of organoid growth medium was gently added to each well.

### 2.3. Passage and Cryopreservation of Bovine Intestinal Organoids

Bovine intestinal organoids were subjected to passage approximately once a week upon maturation. Briefly, the medium was gently aspirated and rinsed with ice-cold PBS without disturbing the organoid dome. To harvest the organoids, a 10× volume of enzyme-free cell disassociation buffer (1 mL) was added to a Matrigel dome (100 μL) in each well and incubated for 10 min in an incubator. Organoids were dislodged by gentle pipetting and collected by centrifugation at 300× *g* for 5 min. The pellet was resuspended in the desired amount of medium and Matrigel in a 1:1 ratio, and each well (140–150 organoids) was distributed into three parts in subsequent passages and seeded in 24-well plates. The medium was replaced every 3 days and sub-cultivated once a week. The number of organoids was recorded in triplicate every week. For cryopreservation, organoids were resuspended in a preserving solution composed of 90% medium and 10% dimethyl sulfoxide (Sigma-Aldrich, Saint Louis, MO, USA), stored at −80 °C for 24 h and transferred to a liquid nitrogen tank for long-term storage.

### 2.4. Histology and Immunohistochemistry

The small intestine segments were fixed in 10% neutral-buffered formalin (Sigma-Aldrich) after strong washing with ice-cold PBS. The segments were subsequently embedded in a paraffin block, and the paraffin-embedded intestinal tissue was vertically and horizontally sectioned at a thickness of 3–5 μm. The sections were then deparaffinised in xylene, rehydrated with water via a graded alcohol series, and processed prior to haematoxylin and eosin (Merck, Darmstadt, Germany) staining. For the immunohistochemical analysis, the sections were permeabilised with 0.1% Triton X-100 in PBS for 5 min and incubated with 0.1% normal goat serum for 1 h to block nonspecific binding after antigen retrieval by boiling the sections in a sodium citrate buffer solution. The samples were incubated with appropriate dilutions of primary antibodies at 4 °C overnight. The antibodies used in the present study are shown in Table S1. After washing, the samples were reacted with anti-mouse and anti-rabbit secondary antibodies coupled to Alexa Fluor-488 and Alexa Fluor-594 (Molecular Probes/Life Technologies, Waltham, MA, USA), respectively, for 1 h at room temperature. These fluorescent samples were counterstained with diamidino-2-phenylindole (DAPI). The images were captured using an Olympus X100 confocal microscope (Olympus, Tokyo, Japan).

### 2.5. Immunocytochemistry

The organoids were maintained in 24-well plates until maturation. The fixation media was aspirated in the wells, and the organoids were washed thoroughly with cold PBS and incubated in neutrally buffered 4% paraformaldehyde (Sigma-Aldrich) for 30 min at room temperature. Then, the organoids were permeabilised in buffer containing 0.5% Triton X-100 (Sigma-Aldrich, Saint Louis, MO, USA) in PBS for 30 min at room temperature. The blocking step was performed using 3% bovine serum albumin in PBS for 1 h at room temperature. The organoids were thoroughly rinsed with PBS and incubated overnight at 4 °C with the appropriate primary antibodies, as shown in Table S1, at their appropriate dilutions. The marker gene expression was detected by incubating the samples with corresponding secondary antibodies coupled to AlexaFluor-488 and AlexaFluor-594 (Molecular Probes/Life Technologies, Carlsbad, CA, USA) for 1 h at room temperature. These fluorescent samples were counterstained with diamidino-2-phenylindole (DAPI) and mounted on glass slides using ProLong Gold Antifade (Life Technologies, Waltham, MA, USA) mounting medium. The images were captured under an Olympus X100 confocal microscope (Olympus, Tokyo, Japan).

### 2.6. Epithelial Barrier Permeability Assay Using FITC-Dextran

Epithelial barrier function was tested by diluting powdered Fluorescein isothiocyanate (FITC)-dextran (4 and 40 kDa) (Sigma-Aldrich, Saint Louis, MO, USA) in nuclease-free water, which resulted in a 1 mg/mL working solution. The organoids were placed in 24-well plates and allowed to grow until fully developed into crypt and villi structures. Then, 25 ng/mL FITC-dextran was added to each well, and the plate was incubated under normal growth conditions. The permeability was observed using luminal absorption and recorded for more than 180 min at 30 min intervals under a Leica CTR6000 fluorescence microscope (Leica, Wentzler, Germany).

### 2.7. RNA Isolation

Total RNA for prepared samples including intestinal organoids was isolated using TRIzol reagent (Life Technologies, Carlsbad, CA, USA), as described previously [25,26]. RNA quality was assessed by an Agilent 2100 bioanalyser using an RNA 6000 nano chip (Agilent Technologies, Amstelveen, The Netherlands), and RNA quantification was performed using an ND 2000 spectrophotometer (Thermo Inc., Wilmington, DE, USA).

### 2.8. Quantitative RT-PCR

Quantitative RT-PCR was performed to assess the expression of several markers of intestinal stem cells and epithelium in bovine intestinal organoids (BIOs) at passage 13 with muscle tissue as a control. Total RNA samples were prepared using TRIzol reagent (Invitrogen, USA). Total RNA (1 µg) was reverse transcribed using the superscript III first-strand synthesis system (Invitrogen, Carlsbad, CA). The PCR reaction mixture was prepared by adding 2 μL PCR buffer, 1.6 μL 2.5 mM dNTP, 10 pmol each forward and reverse primer, 1 μL 20× Eva green, 0.2 μL Taq DNA polymerase, and 2 μL cDNA to a final volume of 20 μL. PCR was performed by means of an initial incubation at 94 °C for 3 min, followed by 40 cycles at 94 °C for 30 s, 60 °C for 30 s, and 72 °C for 30 s, using a melting curve program (increasing temperature from 55 to 95 °C at a rate of 0.5 °C per 10 s) and continuous fluorescence measurement. Sequence-specific products were identified by generating a melting curve. The Ct value represents the cycle number at which a fluorescent signal increases to a level significantly higher than the background, and gene expression was quantified by the 2^−ΔΔCt^ method [27]. qPCR primers for each target gene and 18S ribosomal RNA (rRNA) are listed in Table S2. Gene expression was normalised to that of bovine 18S rRNA. qPCR analysis of mRNAs was performed using the StepOnePlus™ Real-Time PCR System (Applied Biosystems, Foster City, CA, USA).

### 2.9. Library Preparation and Sequencing

For control and test RNAs, library construction was performed using QuantSeq 3′ mRNA-Seq. Library Prep Kit (Lexogen, Inc., Vienna, Austria) was used according to the manufacturer’s instructions. In brief, each 500 ng of total RNA was prepared, an oligo-dT primer containing an Illumina-compatible sequence at its 5′ end was hybridised to the RNA, and reverse transcription was performed. After degradation of the RNA template, a second strand synthesis was initiated by a random primer containing an Illumina-compatible linker sequence at its 5′ end. The double-stranded library was purified using magnetic beads to remove all reaction components. The library was amplified to add the complete adapter sequences required for cluster generation. The finished library is purified from PCR components. High-throughput sequencing was performed as single-end 75 sequencing on a NextSeq 500 (Illumina, Inc., San Diego, CA, USA).

### 2.10. Data Analysis

QuantSeq 3’ mRNA-Seq. reads were aligned using Bowtie2 [28]. Bowtie2 indices were generated from either the genome assembly sequence or the representative transcript sequences for alignment to the genome and transcriptome. The alignment file was used for assembling transcripts, estimating their abundances, and detecting differential expression of genes. Differentially expressed genes were determined based on counts from unique and multiple alignments using the coverage command in Bedtools [29]. The RC (read count) data were processed based on the quantile normalisation method using EdgeR within R (R Development Core Team, Vienna, Austria, 2016) using Bioconductor [30]. Gene classification was based on searches performed in the DAVID (http://david.abcc.ncifcrf.gov, accessed on 15 July 2021) and Medline databases (http://www.ncbi.nlm.nih.gov, accessed on 15 July 2021).

### 2.11. Data Availability

QuantSeq 3′ mRNA-Seq data sets are available via the following accession code in the Gene Expression Omnibus (GEO) database: GSE163425.

### 2.12. Statistical Analysis

Data were analysed by evaluating the differences among treatments using Duncan’s multiple range tests through the general linear model function and Student’s *t*-test of the SAS software (Systat, Cary, NC, USA). The results are expressed as the mean ± standard error (*n* ≥ 3, where *n* is the number of replicates). A *p* value of <0.05 was considered to be statistically significant.

## 3. Results

### 3.1. Long-Term Cultivation of Bovine Intestinal Organoids

**Figure 1:**
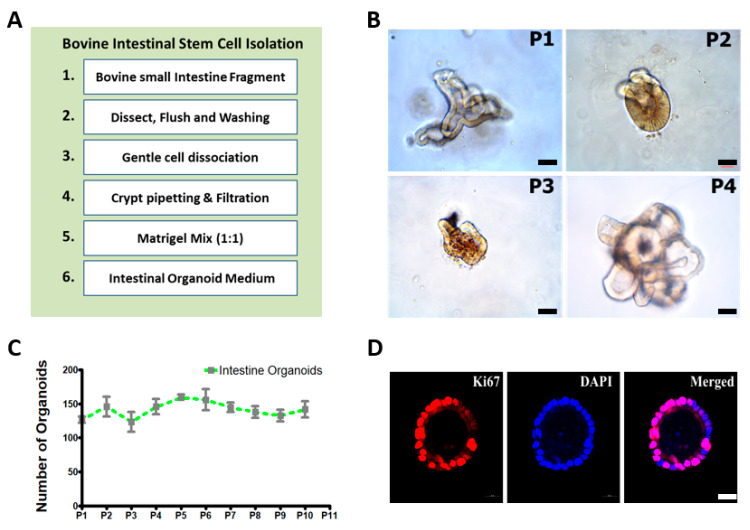
Isolation of intestinal crypts and three-dimensional (3D) cultivation of intestinal crypts including intestinal stem cells in bovine: (**A**) experimental procedures regarding the isolation of intestinal crypts from the small intestine and the three-dimensional (3D) cultivation of intestinal crypts including intestinal stem cells in bovine; (**B**) robust 3D expansion of intestinal crypts including bovine intestinal stem cells (P1–P4). The organoids can be spheroidal (round shaped), show budding (spheroids with extension), and have mature villi and crypt-like structures (branched structures). Scale bar: 50 μm; (**C**) growth rate graph of bovine intestinal organoids showing the number of organoids/well (mean *n* = 3 wells) growing in a 100 μL Matrigel dome in each well. Intestinal organoids were maintained for up to 10 generations without loss of the recapitulating capacity of crypts; (**D**) intestinal organoids were immunostained for Ki67 at passage 5, a marker of proliferating cells, and were counterstained with diamidino-2-phenylindole (DAPI). Scale bar: 20 μm.

Intestinal crypts were isolated from the small intestine (Jejunum) of healthy Hanwoo cattle (>24 months old), sequentially embedded in Matrigel, and cultivated in the IntestiCult medium. Figure 1A illustrates the experimental procedures for the isolation of intestinal crypts and the cultivation of bovine intestinal organoids. The recapitulating capacity of the organoids was demonstrated by the stable growth for more than passage 10 (P10) and the long-term maintenance. As shown in Figure 1B, these organoids showed various morphologies such as spheroidal (round shaped), budding (spheroids with extension), and mature villi and crypt-like structures from P1 to P4 generations at early passages. Subsequent detailed structures and development of intestinal organoids at each passage represent the spheroidal structure from day 0 to the fully grown structure on day 8 after isolation and cultivation of intestinal crypts from the small intestine (Supplementary Figure S1). Furthermore, they showed consistent growth in an average of 130–150 organoids per basement matrix dome from P1 to P10 at each generation, indicating the recapitulating capacity of the crypt (Figure 1C). At this density, bovine intestinal organoids showed continuous proliferation and growth. Furthermore, intestinal organoids at P5 were identified as positive against Ki67, a proliferating cell marker (Figure 1D). Collectively, these results demonstrated that bovine intestinal organoids cultivated and isolated from small intestine (Jejunum) crypts were maintained long-term without loss of the recapitulating capacity of crypts.

### 3.2. Identification of Intestinal Stem Cells from Bovine Small Intestine

**Figure 2:**
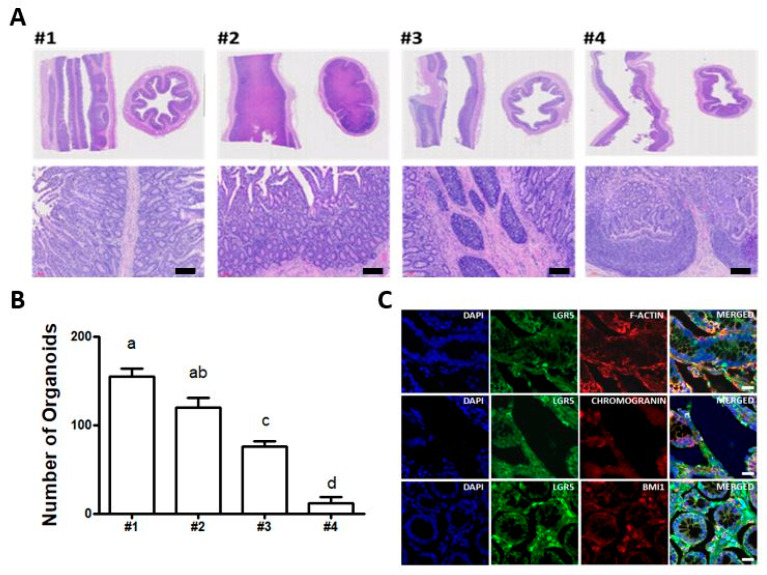
Immunohistochemical analysis of bovine small intestine: (**A**) haematoxylin and eosin histological staining to identify distinct crypt and villus structures from four different locations (#1, #2, #3, and #4) in the jejunum between the duodenum and ileum. Scale bar: 300 μm; (**B**) the number of intestinal organoids per basement matrix dome to verify the efficiency of the derivation of intestinal organoids from four different locations. The number of intestinal organoids derived from location #1 in the jejunum close to the duodenum was significantly higher, compared to locations #3 and #4. The values are the means plus the standard error of mean (S.E.M) and different letters (a–d) indicate significant differences (*p* < 0.05); (**C**) immunohistochemistry of LGR5, Bmi1, F-actin, and Chromogranin A in bovine small intestine. The fluorescently stained crypts were counterstained with diamidino-2-phenylindole (DAPI). Scale bar: 20 μm.

To search for position effects for efficient isolation and cultivation of intestinal organoids from the small intestine, we selected four different locations in the jejunum between the duodenum and ileum. Initially, intestinal tissue sections were subjected to anatomical analysis using haematoxylin and eosin histological staining to identify distinct crypt and villus structures. As shown in Figure 2A, locations #1 and #2 were better developed than locations #3 and #4. The detailed view from vertical and horizontal sections in locations #1 and #2 showed integral structures of the intestinal epithelium gland, such as crypts at the bottom and finger-shaped villi on the apical side (Supplementary Figure S2). Furthermore, to verify the efficiency of the derivation of intestinal organoids from four different locations, we subsequently cultivated intestinal organoids. Based on the results, the number of intestinal organoids per basement matrix dome was highest in location #1 (Figure 2B), indicating the most growth potential for the derivation of intestinal organoids. In addition, to identify intestinal stem cells in vivo, immunohistochemistry with respect to several markers involved in intestinal stem cells and epithelial cells was conducted. As shown in Figure 2C, intestinal crypts isolated from location #1 of the small intestine had a distinct expression, such as leucine-rich repeat-containing G protein-coupled receptor 5 (LGR5), a key gene required for stemness that is expressed in columnar crypt cells, B lymphoma Mo-MLV insertion region 1 homology (Bmi1), which was found in +4 cells adjacent to Paneth cells and F-actin in the intestinal epithelial cytoskeleton. Moreover, the fluorescently stained crypts showed epithelium-specific expression of Mucin2 in goblet cells, E-cadherin in adherent junctions (Supplementary Figure S3). Together, these results are the first to show the identification of LGR5^+^ intestinal stem cells from the small intestine and demonstrate the regional differences for efficient generation of intestinal organoids in bovine.

### 3.3. Characterisation and Paracellular Permeability of Bovine Intestinal Organoids

**Figure 3:**
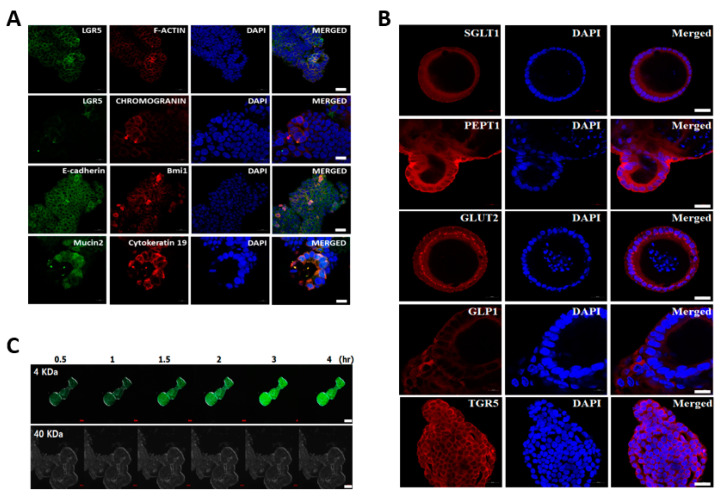
Characterisation and paracellular permeability of bovine intestinal organoids: (**A**) immunostaining of LGR5, Bmi1, F-actin, E-cadherin, Chromogranin A, and Mucin2 in bovine intestinal organoids at passage 5. The organoids were counterstained with DAPI. Scale bar: 20 μm; (**B**) immunostaining of several representative nutrient absorption markers such as sodium-dependent glucose transporter (SGLT1), proton-coupled peptide transporter (PEPT1), glucose transporter (Glut2), glucagon-like peptide 1 (GLP1), and bile acid receptor (TGR5) in bovine intestinal organoids at passage 5. The organoids were counterstained with DAPI. Scale bar: 20 μm; (**C**) paracellular permeability of the epithelial layer in bovine intestinal organoids using fluorescent tracers. Intestinal organoids were treated with FITC-dextran to assess barrier function. FITC-dextran 4 kDa showed high permeability, while FITC-dextran 40 kDa failed to enter the organoid lumen. Scale bar: 20 μm.

To characterise the cellular potentials of bovine intestinal organoids derived from the small intestines of adults, we investigated the spatial expression of several specific markers involved in intestinal stem cells and epithelium characteristics in bovine intestinal organoids at passage 5. As shown in Figure 3A, the organoids had a distinct expression, such as LGR5 and Bmi1. Moreover, the fluorescently stained organoids showed epithelium-specific expression against Mucin2 for goblet cells that contributes to epithelial barrier integrity, E-cadherin for adherent junctions, F-actin for intestinal epithelial cytoskeleton, Chromogranin A for enteroendocrine cells, and Cytokeratin 19 for enterocytes, indicating that the concomitant expression of intestinal epithelial genes in intestinal organoids derived from intestinal crypts mimicked the topology of an in vivo intact intestine. Furthermore, intestinal organoids at P5 were immunoreactive to antibodies against several representative nutrient absorption markers, especially sodium-dependent glucose transporter (SGLT1), proton-coupled peptide transporter (PEPT1), glucose transporter (Glut2), glucagon-like peptide 1 (GLP1), and bile acid receptor (TGR5) (Figure 3B). In addition, we investigated the paracellular permeability character of the epithelial layer using fluorescent tracers up to 4 hr after treatment. FITC-dextran labelled the organoid lumen, demonstrating a high permeability for compounds of up to 4 kDa, such as glucose, peptides, and fatty acids, while FITC-dextran 40 kDa failed to enter the organoid lumen (Figure 3C). The concentration of FITC was maintained constantly and slow diffusion of FITC-dextran 4 kDa start to enter the organoid lumen at 1.5 h (Supplementary Figure S4), indicating the presence of a mucous layer, which plays a major role in barrier function and nutrient absorption. The mucosal barrier function results showed that there was mucins secretion by goblet cells. However, it seems likely that FITC-dextran 4 kDa did not reach the apical surface due to the basal-out structure of bovine intestinal organoids. Together, these functional testing results suggested that intestinal organoids had physiological relevance to the in vivo gut absorption properties.

### 3.4. Gene Expression Profiling of Bovine Intestinal Organoids

**Figure 4:**
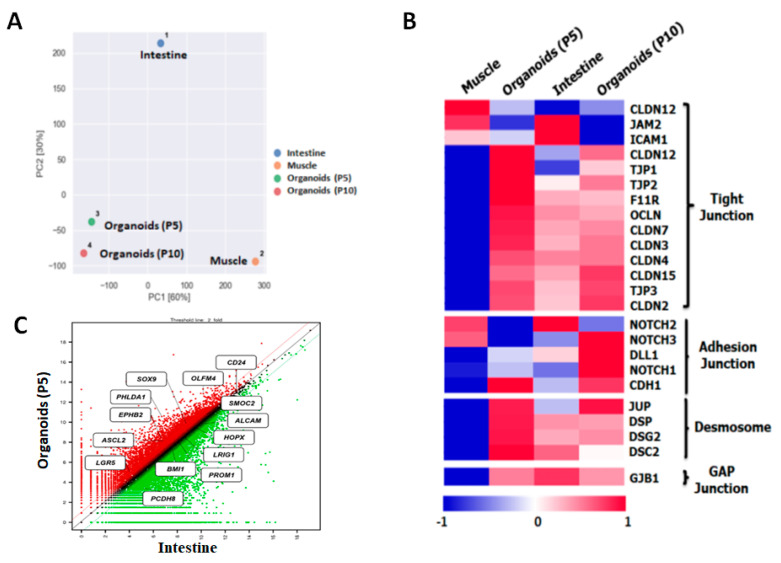
Gene expression profiling of bovine intestinal organoids using QuantSeq 3′ mRNA-Seq. Library: (**A**) principal component analysis (PCA) of intestinal organoids (P5), intestinal organoids (P10), small intestine, and muscle in bovine. The distance between intestinal organoids and the small intestine was relatively close compared to muscle; (**B**) heatmap showing many genes in epithelium-characteristic categories such as tight junctions, adherent junctions, desmosomes, and gap junctions. Intestinal organoids at P5 and P10 and in the small intestine were significantly expressed compared to muscle as a control; (**C**) scatter plot showing many genes related to intestinal stem cell markers such as *LGR5*, *ASCL2*, *EPHB2*, *PHLDA1*, *SOX9*, and *OLFM4*. Intestinal organoids at P5 were significantly upregulated or similar between intestinal organoids and the intestine.

**Figure 5:**
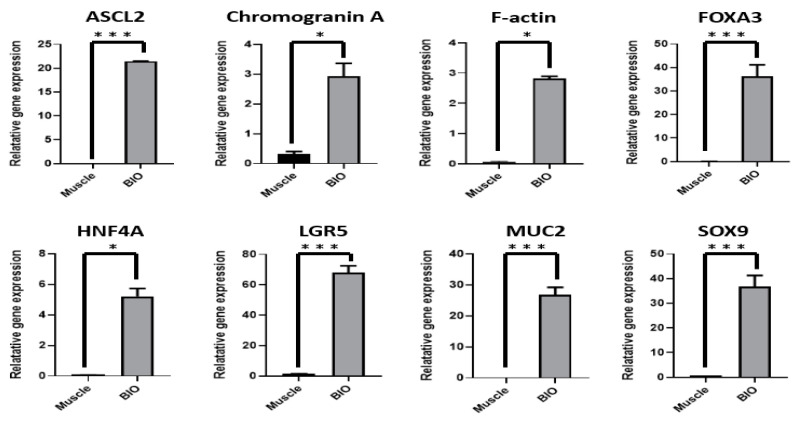
Gene expression profiling of bovine intestinal organoids using quantitative RT-PCR. Quantitative RT-PCR was performed to evaluate gene expression of bovine intestinal organoids (BIO) using several markers of intestinal stem cells (*LGR5*, *ASCL2*, *HNF4A*, *FOXA3*, and *SOX9*) and epithelium (*MUC2*, *Chromogranin A*, and *F-actin*) characteristics with muscle from adult bovine as a control. Gene expression was normalised to that of 18S rRNA and analysed by the 2^−ΔΔCt^ method. Significant differences between groups were analysed by Student’s *t*-test. A *p* value less than and equal to 0.05 indicated statistical significance (* *p* value ≤ 0.05, ** *p* value ≤ 0.01, *** *p* value ≤ 0.001).

To investigate the genetic properties of bovine intestinal organoids for large-scale gene expression profiling, QuantSeq 3′ mRNA-Seq. library was constructed. As shown in Figure 4A, principal component analysis (PCA) indicated that the distance between intestinal organoids and the small intestine was relatively close, compared to muscle in bovine. In addition, the heatmap showed that many genes in epithelium-characteristic categories, such as tight junctions, adherent junctions, desmosomes, and gap junctions, were significantly expressed in intestinal organoids at P5 and P10 and in the small intestine, compared to muscle as a control (Figure 4B). Furthermore, scatter plot revealed that many genes related to intestinal stem cell markers such as *LGR5*, achaete-scute family BHLH transcription factor 2 (*ASCL2*), EPH receptor B2 (*EPHB2*), pleckstrin homology like domain family a member 1 (*PHLDA1*), SRY-box transcription factor 9 (*SOX9*) and Olfactomedin 4 (*OLFM4*) were significantly upregulated in intestinal organoids at P5 or similar between intestinal organoids and the intestine (Figure 4C). Moreover, to validate QuantSeq 3′ mRNA-Seq. data and evaluate gene expression of bovine intestinal organoids (BIO) using a set of genes involved in intestinal stem cell (*LGR5*, *ASCL2*, hepatocyte nuclear factor 4 alpha (*HNF4A*), forkhead box A3 (*FOXA3*) and *SOX9*), and epithelium (*MUC2*, *Chromogranin A* and *F-actin*) with muscle from adult bovine as a control, quantitative RT-PCR was conducted. As shown in Figure 5, the intestinal stem-cell-related genes such as *LGR5* (*p* < 0.001), *ASCL2* (*p* < 0.001), *HNF4A* (*p* < 0.05), *FOXA3* (*p* < 0.001) and *SOX9* (*p* < 0.001) and intestinal epithelium such as *MUC2* (*p* < 0.001), *Chromogranin A* (*p* < 0.05), and *F-actin* (*p* < 0.05) were significantly higher on intestinal organoids than muscle. Taken together, these results indicated that the genetic properties of bovine intestinal organoids were highly similar to those of in vivo.

## 4. Discussion

Recently, intestinal organoids have evolved as potential alternatives to in vivo systems [11,31] and have been a focus of research in livestock species including bovine, swine, and chicken [9,10,18,32]. Especially, these organoids have been shown to be an attractive model for mucosal permeability, enabling us to investigate the interactions of pathogenic bacteria, viruses, nutrient absorption, and the maintenance of host homeostasis with the gut epithelium of their host [33,34,35,36].

In the present study, we successfully established intestinal organoids derived intestinal crypts from the small intestine and reported establishment and robust expansion of intestinal organoids in bovine. Firstly, we isolated intestinal crypts that included intestinal stem cells from the small intestine (jejunum) in adult bovines and cultivated them using the scaffold-based method. The organoids representing distinct crypt and villus structures surrounding the lumen can be long-term maintained (>P10) during several passages without loss of their recapitulating capacity. Furthermore, we achieved a consistent growth rate of 130–150 per basement matrix dome and identified it as positive against Ki67, a proliferating cell marker [19] in intestinal organoids at P5 (Figure 1). The results of the current study suggest that the organoids closely mimicked the in vivo organ physiology and remained indefinitely intact under controlled conditions without loss of the recapitulating capacity of crypts.

Next, to assess the position effects for efficient isolation and derivation of intestinal organoids along the length of the small intestine in bovine, we sectioned four different locations in the jejunum between the duodenum and ileum. Interestingly, we found that jejunum (location #1 and #2) close to the duodenum showed integral structures of the intestinal epithelium gland, such as crypts at the bottom and finger-shaped villi on the apical side and intestinal crypts isolated from location #1 in the jejunum close to the duodenum had the most growth potential for the derivation of intestinal organoids (Figure 2). However, mouse models have shown that more distal tissues in the foetal intestine can be formed organoids well [37], suggesting the regional differences in vitro growth potential. Therefore, it seems likely that such information would be valuable for the derivation of intestinal organoids and subsequent robust expansion due to physiological differences between species or ages. In addition, in search for detailed identification of intestinal stem cells from the small intestine in bovine, we confirmed that the intestinal crypts isolated from the small intestine in bovine had the distinct expression of leucine-rich repeat-containing G protein-coupled receptor 5 (LGR5, a key gene required for stemness and expressed in columnar crypt cells), B lymphoma Mo-MLV insertion region 1 homology (Bmi1, which was found in +4 cells adjacent to Paneth cells), F-actin in the intestinal epithelial cytoskeleton, epithelium-specific expression of Mucin2 in goblet cells and E-cadherin in adherent junctions, supporting previous studies on mouse and human intestinal organoids such as the mini-gut [16,19]. Collectively, our findings demonstrate for the first time that the identification of LGR5^+^ intestinal stem cells from the small intestine and demonstrate the regional differences for efficient derivation of intestinal organoids in bovine.

With regard to the cellular potentials of bovine intestinal organoids, we investigated the several specific markers involved in intestinal stem cells and epithelium’s characteristics. As shown in Figure 3, bovine intestinal organoids had a distinct expression, such as LGR5 and Bmi1 for self-renewal capacities, and also showed epithelium-specific expression against Mucin2, indicating the presence of mucin secreting in goblet cells that lined up the epithelial mucosa, E-cadherin for adherent junctions, F-actin for intestinal epithelial cytoskeleton, Chromogranin A for enteroendocrine cells and Cytokeratin 19 for enterocytes representing the cellular diversity composed of the intestinal epithelium. It is well accepted that intestinal organoids consist of several types of intestinal cells including intestinal stem cells, Paneth cells, enteroendocrine cells, goblet cells, transit-amplifying cells, and enterocytes [2]. Based on our results, the cellular potentials of intestinal organoids derived from the small intestines of adult bovine relatively resemble in vivo small intestines and are similar to that of human intestinal organoids [38]. In addition, the intestinal epithelium plays an important role in nutrient absorption across the membrane and the diffusion of small molecules across the intestinal barrier, thus ensuring health by nutrient absorption and preventing bacterial translocation via the bloodstream [39,40]. Therefore, we further investigated the several representative nutrient absorption markers and paracellular permeability character of the epithelial layer [41]. As shown in Figure 3B,C, we found that intestinal organoids at P5 had the specific-expression of sodium-dependent glucose transporter (SGLT1), proton-coupled peptide transporter (PEPT1), glucose transporter (Glut2), glucagon-like peptide 1 (GLP1), and bile acid receptor (TGR5), and showed a high permeability for compounds of up to 4 kDa, such as glucose, peptides, and fatty acids, while FITC-dextran 40 kDa failed to enter the organoid lumen, indicating the absorptive capacity of bovine intestinal organoids. Taken together, these results indicate that bovine intestinal organoids preserve the functional and phenotypic characteristics of the small intestine and can be used as physiological indicators of nutrient absorption in nutritional research showing mimicked the topology of an in vivo intact intestine.

With regard to the genetic properties of bovine intestinal organoids using large-scale gene expression profiling and quantitative RT-PCR, we found that the distance between intestinal organoids and the small intestine was relatively close, compared to muscle in bovine and many genes in epithelium-characteristic categories [36], such as tight junctions, adherent junctions, desmosomes, and gap junctions, were significantly expressed in intestinal organoids at P5 and P10 (Figure 4), suggesting the physiological similarity between the intestinal organoids and small intestine. In addition, they had a significant expression on many genes related to intestinal stem cells such as *LGR5*, *ASCL2*, *HNF4A*, *FOXA3*, and *SOX9* and intestinal epithelium such as *MUC2*, *Chromogranin A*, and *F-actin* (Figure 5). Collectively, our findings suggested that the genetic properties of bovine intestinal organoids were highly similar to those of in vivo.

## 5. Conclusions

These results provide a reliable method for efficient isolation of intestinal crypts from the small intestine and robust 3D expansion of intestinal organoids in adult bovine and demonstrate that the in vitro 3D organoids mimic the in vivo tissue topology and functionality. Finally, intestinal organoids are potential alternatives to in vivo systems and will facilitate the practical use of a model to replace animal experiments in the fields of animal biotechnology for various purposes.

## Data Availability

The datasets during and/or analysed during the current study available from the corresponding authors on reasonable request.

## Supplementary Materials

The following are available online at https://www.mdpi.com/article/10.3390/ani11072115/s1, Figure S1: Three-dimensional (3D) cultivation of intestinal crypts including intestinal stem cells isolated from small intestine in bovine. Intestinal organoids show various developmental stages and can be spheroidal (round shaped), show budding (spheroids with extension), and have mature villus and crypt-like structures (branched structures). The detailed structures represent organoid propagation from day 2 to the fully grown structure on day 8. Scale bar: 100 μm (day 0, day 2) and 50 μm (day 3, day 6, and day 8), Figure S2: Histological H and E staining of the vertical and horizontal views reveal the positions of the intestinal epithelial glands, such as crypts (oval structures at the bottom) and villi (finger-like structures) cells, with varying magnification (40×, 100×, and 200×) at four different locations from #1 to #4. The purple spots show nuclei and the epithelium is stained pink, Figure S3: immunohistochemistry of Bmi1, E-cadherin, Chromogranin A, and Muccin2 in bovine small intestine. The fluorescently stained crypts were counterstained with diamidino-2-phenylindole (DAPI). Scale bar: 20 μm, Figure S4: real-time monitoring and slow diffusion of FITC-dextran 4 kDa to enter the organoid lumen. Scale bar: 20 μm, Table S1: antibodies used in the present study for characterisation of bovine intestinal organoids, Table S2: primers used for the gene expression analysis of bovine intestinal organoids.
