# Supplementary material for: Robust Three-Dimensional (3D) Expansion of Bovine Intestinal Organoids: An In Vitro Model as a Potential Alternative to an In Vivo System
Source: Animals (Basel). 2021 Jul 16;11(7):2115. doi: 10.3390/ani11072115 (PMC8300217; doi:10.3390/ani11072115)
Supplement: Supplementary file 1 [file animals-11-02115-s001.zip › Table S1_20210628_Revised.pdf]

Table S1. Antibodies used in this study for characterization of bovine intestinal organoids.

Antibody	Host species	Dilution	Company (Catalog No.)
LGR5	Mouse	1:50	Origene Technologies, Inc. (TA503316)
Bmi1	Rabbit	1:50	abcam (ab38295)
Mucin2	Mouse	1:50	Santa Cruz Biotechnology, Inc. (SC-515032)
E-Cadherin	Mouse	1:200	BD Biosciences (61081)
Cytokeratin 19	Rabbit	1:200	abcam (ab84632)
F-actin	Rabbit	1:200	abcam (ab83746)
Chromogranin A	Rabbit	1:200	abcam (ab85554)
Glut2	Rabbit	1:100	Novus Biologicals (NBP1-69466)
PEPT1	Rabbit	1:100	Bioss Antibodies (BS-0689R)
SGLT1	Rabbit	1:100	Novus Biologicals (NBP2-20338)
GLP1	Rabbit	1:100	MyBioSource (MBS2107860)
TGR5	Rabbit	1:100	abcam (ab72608)
Ki67	Rabbit	1:200	Cell Signaling Technology (D3B5)
