# Supplementary material for: Robust Three-Dimensional (3D) Expansion of Bovine Intestinal Organoids: An In Vitro Model as a Potential Alternative to an In Vivo System
Source: Animals (Basel). 2021 Jul 16;11(7):2115. doi: 10.3390/ani11072115 (PMC8300217; doi:10.3390/ani11072115)
Supplement: Supplementary file 1 [file animals-11-02115-s001.zip › Table S2_20210628_Revised.pdf]

Table S2. Primers used for the gene expression analysis of bovine intestinal organoids.

Gene Name	Forward	Reverse
18S rRNA	AAACGGCTACCACATCCAAG	CCTCCAATGGATCCTCGTTA
HNF4A	CGAGCAGATCCAGTTCATCA	GAAGGCTGTGGAGTCTCAGG
FOXA3	GCAAGATGCTTACCCTGAGC	AGGTAGCAGCCGTTCTCAAA
ASCL2	AGTTGAGCAAGGTGGAGACG	CCGACGAGTAGGCGGAAC
SOX9	TTCATGAAGATGACCGACGA	GTCCAGTCGTAGCCCTTGAG
LGR5	GTGTTCAGAGCCGCAGTGTA	GATTCCGAAGCAAAAATGGA
MUC2	TTCGACGGGAGGAAGTACAC	TTCACCGTCTGCTCATTCAG
Chromogranin A	TCTCAATCCTGCGACATCAG	CTGTCTCCGTCCGAGTCTTC
F-actin	AATCAGAGGCCAAGGGAACT	TGCAGGATGAGCTTGTTGTC
